# Nearly Complete Genome Sequence of a Sapelovirus A Strain Identified in Swine in Italy

**DOI:** 10.1128/MRA.00481-19

**Published:** 2019-07-18

**Authors:** L. Tassoni, G. Zamperin, I. Monne, M. S. Beato

**Affiliations:** a Diagnostic Virology Laboratory, Istituto Zooprofilattico Sperimentale delle Venezie, Legnaro, Padua, Italy; b Research and Development Laboratory, Istituto Zooprofilattico Sperimentale delle Venezie, Legnaro, Padua, Italy; Portland State University

## Abstract

We report the first nearly complete genome sequence of a porcine sapelovirus (PSV) A strain that was identified from feces of piglets suffering from diarrhea in Italy in 2015. Phylogenetic investigations revealed a separate clustering for the Italian PSV, indicating unique molecular features.

## ANNOUNCEMENT

Sapelovirus A, formerly known as porcine sapelovirus (PSV), is a single-stranded positive-sense nonenveloped RNA virus of the genus Sapelovirus and family *Picornaviridae*. The large open reading frame (ORF) encodes a single polyprotein subsequently processed into 4 structural proteins (VP1 to VP4), with a leader peptide (L) at the N terminus and 7 functional proteins (2A, 2B, 2C, 3A, 3B, 3C, and 3D). This virus was reported in asymptomatic (1) and diseased swine presenting with a wide variety of symptoms, such as diarrhea, respiratory distress, reproductive failure, and polioencephalomyelitis (2, 3), in 3 regions, America, Asia, and Europe (2, 4–9). The Italian PSV herein described was identified in feces collected from a piglet suffering from acute gastroenteritis and diarrhea in an open-cycle farm in Northeast Italy in November 2015.

Total RNA was extracted using the QIAamp viral RNA minikit (Qiagen, Hilden, Germany), according to the manufacturer’s instructions, investigated by a next-generation sequencing (NGS)-based metagenomics approach using the TruSeq stranded total RNA kit with Ribo-Zero Gold, and processed on a HiSeq instrument with the HiSeq reagent kit v4 (2 × 125-bp paired-end [PE] mode; Illumina, San Diego, CA, USA).

Sequencing yielded 53,586,949 paired-end reads that were 125 bp long, which were quality filtered and taxonomically classified by (i) aligning against the integrated NT database (version 8, February 2017) using BLAST 2.6.0+ (10), with default parameters, and against the integrated NR database (version 8, February 2017) using DIAMOND version 0.8.36 (11), with default parameters; (ii) filtering out alignment hits with an E value larger than 1 × 10^−3^; and (iii) feeding remaining alignment hits to MEGAN ultimate edition version 6.7.0 (12). Reads taxonomically classified as belonging to the sapelovirus A species were selected and *de novo* assembled using IDBA-UD version 1.1.1 (13), using default parameters and the multi-*k*-mer approach (minimum value, 24; maximum value, 124; increment, 10). A single contig with a length comparable to the PSV genome size was obtained. All reads belonging to the sapelovirus A species were subsequently aligned against the longest contig from the *de novo* assembly using BWA version 0.7.12 (14), with standard parameters. The alignment was manually revised with Tablet (15) to verify that all nucleotides were the consensus ones, verify the absence of misaligned reads, and avoid the risk of misassembly. The final average coverage of the Sapelo_A_Italy/DIAPD5469-10/2015 genome was 568-fold.

The first nearly complete genome sequence of an Italian PSV strain (Sapelo_A_Italy/DIAPD5469-10/2015) was 7,564 nucleotides (nt) long.

The genome contained a 7,014-nt-long ORF encoding a putative 2,338-amino acid (aa) polyprotein, preceded by a 475-nt 5′ untranslated region (UTR) and followed by a 75-nt 3′ UTR.

Fifty-one complete sapelovirus genomes are available in public databases, with none from Italy and only four from Europe (as of January 2019).

**FIG 1:**
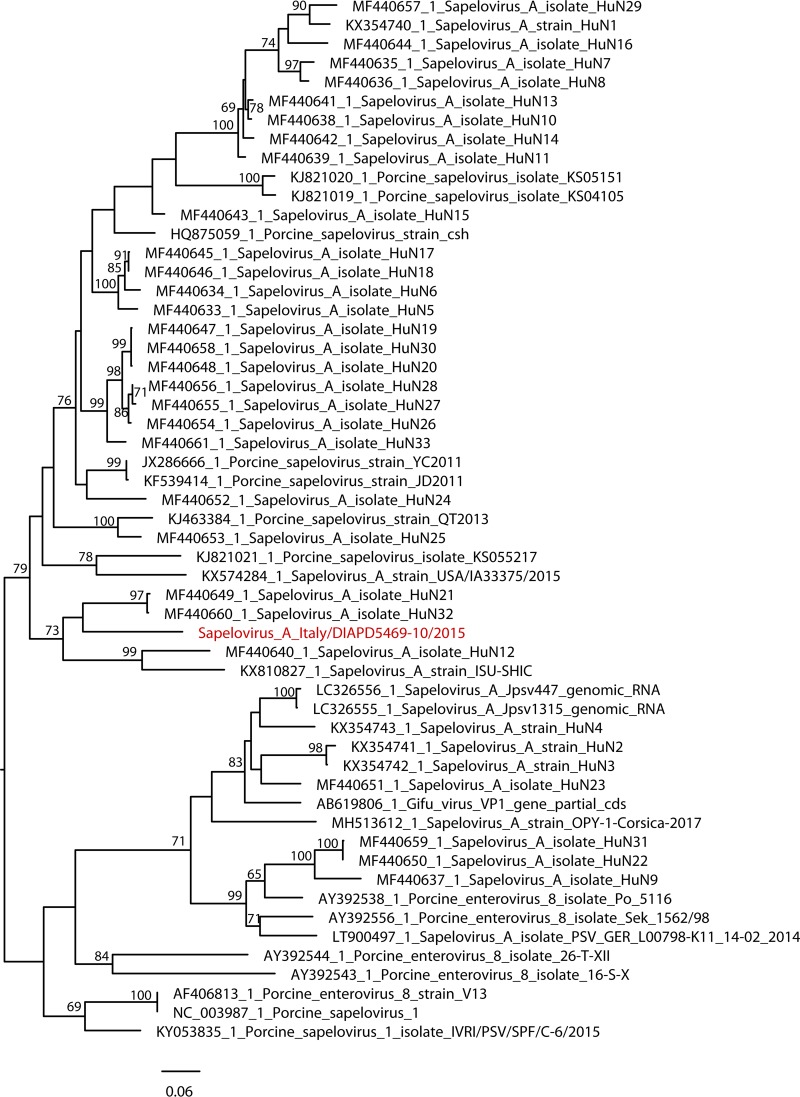
VP1 nucleotide phylogenetic tree. The VP1 nucleotide sequences of swine sapeloviruses were aligned using MEGA 6.0, and phylogeny was inferred by the maximum likelihood method implemented in PhyML 3.0. The applied substitution model is GTR+G+I, obtained using the model finder implemented in MEGA 6.0. A nonparametric bootstrap analysis with 100 replicates was performed to obtain branch supports; only values of ≥60% are shown. The Italian Sapelo_A_Italy/DIAPD5469-10/2015 strain is colored red.

The nucleotide identities of Sapelo_A_Italy/DIAPD5469-10/2015 with the full-length sequences available range from 84.05 to 86.62% and from 94.08 to 96.05% with partial Italian PSV sequences. The phylogenetic investigations of the amino acid and nucleotide sequences of the VP proteins (Fig. 1) and the entire polyprotein revealed a distinctive clustering of the Italian strain, indicating that the circulation of PSVs with unique molecular features is likely.

### Data availability.

HiSeq raw data were submitted to the NCBI Sequence Read Archive (SRA) under accession number SRR6297822. The nucleotide sequence of Sapelovirus_A_Italy/DIAPD5469-10/2015 is deposited in GenBank under accession number MK497044.
